# Unpredictable warm spells in winter increase blood cortisol level but lengthen telomeres in a seasonal rodent *Phodopus sungorus*

**DOI:** 10.1098/rspb.2025.0819

**Published:** 2025-06-18

**Authors:** Hanna Kletkiewicz, Krzysztof Kowalski, Jacek Kęsy, Paulina Trzeciak, Anna Nowak, Małgorzata Jefimow, Anna Przybylska-Piech

**Affiliations:** ^1^ Department of Animal Physiology and Neurobiology, Nicolaus Copernicus University, Toruń, Poland; ^2^ Department of Vertebrate Zoology and Ecology, Nicolaus Copernicus University, Toruń, Poland; ^3^ Department of Plant Physiology and Biotechnology, Nicolaus Copernicus University, Toruń, Poland; ^4^ Department of Ecology and Biogeography, Nicolaus Copernicus University, Toruń, Poland

**Keywords:** stress, cortisol, telomeres, climate change, winter phenotypes, *Phodopus sungorus*

## Abstract

Global warming and the increased frequency of unpredictable weather events may disrupt the proper timing of seasonal adjustments of a phenotype. This may lead to the deterioration of the animal’s condition and shorten its lifespan. We tested whether warm spells in winter affect the baseline and stress-induced cortisol level and leukocyte relative telomere length in two winter phenotypes of Siberian hamster, *Phodopus sungorus*, responding and non-responding to short photoperiod. We found that both phenotypes increased cortisol levels in winter and that warm spells augmented this response. Under stable cold conditions, non-responding individuals were more vulnerable to short-term stress than responding ones. However, telomere length increased, suggesting that animals have a high potential to cope with stress and prevent telomere shortening or that these two variables are not directly related. In responding individuals, the higher incidence of torpor also prevented telomeres shortening. These results indicate that both phenotypes, responding and non-responding to short photoperiod, can overcome the challenges posed by an unpredictably changing environment.

## 1. Introduction

Animals anticipate the forthcoming season, usually based on changes in photoperiod and ambient temperature (*T*_a_), and change their phenotype to match the environment. In winter, when keeping a positive energy balance is more difficult, an imbalance may occur, leading to increased glucocorticoid hormone (GC) levels [1]. Among mammals, most species show an annual rhythm of GC levels, but there is no consensus season when GC is elevated [2]. The 'energy mobilization hypothesis’ posits that GC (cortisol or corticosterone) levels are high during periods of high energy demands because they suppress energy storage and promote energy utilization from adipose tissue and the liver, ensuring the energy supply necessary to cope with stress. This would be in accordance with the results of a meta-analysis showing that the variations in GC levels reflect variations in metabolic rate instead of stress load [3]. Prolonged stress, which induces an increase in GC levels, may also negatively affect antioxidant defence, resulting in oxidative stress, and ultimately, it may lead to a faster telomere attrition rate ([4]; reviewed in [5]). However, the relationship between telomere length and GC levels is inconsistent between species, sexes or locations (reviewed in [5]). The link between oxidative stress and telomere length *in vivo* is also equivocal ([6]; reviewed in [7]). Relative telomere length (RTL) is considered a marker of biological, not chronological age, while its dynamics are an outcome of telomere loss and renewal ([8–11]; reviewed in [5]). Although a meta-analysis of the RTL and age showed only a weak negative correlation [12], telomeres shorten faster in more demanding environments ([9,13]; reviewed in [5]) and in fast-ageing than in slow-ageing animals [14]. Thus, winter as an energy-demanding period should lead to increased GC levels and telomere shortening in small endothermic animals.

Environmental cues that allow animals to anticipate winter can be disrupted by global warming and the increased frequency of unpredictable weather events. According to the Intergovernmental Panel on Climate Change *Climate change 2023: synthesis report* [15], global surface temperature over land was 1.59°C higher in 2011−2020 than in 1850−1900. The freeze-free periods in most mid- and high-latitude regions are lengthening, and snow cover has decreased by 10% since the late 1960s [16]. However, in the ecological context, unpredictable short-term events are more relevant to animal performance than the average global warming [16]. Warm spells can appear unpredictably in summer and winter, posing a threat to heterothermic species that use daily torpor and/or hibernation. These two states of hypometabolism with low body temperatures ensure energy saving but differ in depth and duration, with shallow daily torpor lasting less than 24 h and hibernation lasting for weeks or months (reviewed in [17]). Warm spells in summer may affect reproduction [18,19] or lead to dehydration and life-threatening hyperthermia [20]. In winter, warm spells may disrupt the torpor patterns [21,22], but also change the proportion of different winter phenotypes among individuals [22–25].

We tested the hypothesis that the effects of unpredictable warm spells in winter on animal performance depend on the winter phenotype. Siberian hamster is a perfect model species because it is a heterothermic rodent with a high degree of phenotypic polymorphism. It inhabits the Siberian steppe, where summer ambient temperatures often reach over +30°C while winters are cold [26]. However, global warming also affects this environmental niche. Since 1900, the mean annual temperature in Kazakhstan increased by 2.3°C (https:// climateknowledgeportal.worldbank.org/country/kazakhstan/climate-data-historical). Two extreme winter phenotypes of the Siberian hamster, responding and non-responding to shortening photoperiod, differ considerably in multiple traits that enhance energy conservation or diminish energy loss [27–29]. In winter, responding hamsters enter daily torpor and have smaller body mass (*m*_b_) than in summer, show gonadal regression and change fur from grey to white, while non-responders do not show these changes [27–29]. There is also a group of ‘partial-responders', which develop only some traits of the winter phenotype [22,30,31]. Polymorphism of winter phenotype is observed in many species. In our outbred colony of Siberian hamsters, the proportion of non-responders varies between 35 and 75% [30,32]. It reaches up to 80% in the prairie voles, *Microtus ochrogaster* [33], 47% in Turkish hamsters, *Mesocricetus brandti* [34], 50% in white-footed mice, *Peromyscus leucopus* [35], and 25% in deer mice, *Peromyscus maniculatus* [36]. Despite the obvious benefits of being a responder, non-responders, which express summer phenotype throughout the year, are continuously present in the population ([25,37]; reviewed in [38]).

We predicted that both phenotypes, responding and not responding to short photoperiod, will increase blood cortisol levels in winter because low *T*_a_ can be perceived as stress and increase metabolic rate ([39]; reviewed in [40]). This seasonal change would reflect the predictive homeostasis of the reactive scope model [41], which describes daily and seasonal changes in the mediators of stress in response to predictable and unpredictable changes in the environment. We also predicted that this increase would be smaller in responders than in non-responders because of their morphological and physiological winter adaptations, which can reduce cold-induced increase in GC hormones [1,40,42]. However, we expected that responders would be more sensitive to warm spells in winter because they are less flexible than non-responders [43]. The response to short-term stress would reflect the reactive homeostasis of the reactive scope model [41]. In other words, the predictive homeostasis range for GC would be lower, but the reactive homeostasis range would be higher in responders than non-responders.

Because stress hormone levels may correlate with telomere length (reviewed in [5,39]), we also predicted that non-responders have a faster telomere attrition rate than responders. The use of torpor exclusively by responders may also contribute to the differences in telomere length dynamics between phenotypes. Previous data showed that telomere length was positively correlated with torpor frequency in the Siberian hamster [44]. Both daily torpor and hibernation affect telomere length, but in a way that depends on ambient and body temperatures, food availability, or the frequency of arousals ([44–46]; reviewed in [47]). Another clue suggesting potential differences in RTL between phenotypes is that female Siberian hamsters that respond to short photoperiods age reproductively slower than non-responders, implying they may live longer [48,49]. Finally, small hibernators (<1.5 kg) live longer than non-hibernators of similar size ([50,51], reviewed in [47]). In the face of a rapidly changing environment, phenotypic polymorphism may enhance survival, giving evolutionary advantages to a population [23]. Because climate change is intensifying, knowledge of the phenotypic responses to changing environments is crucial for understanding seasonal variations in health and physiological performance in organisms living in seasonal environments.

## 2. Material and methods

### (a) General procedure

All experiments were done on Siberian hamsters acclimated to summer-like and winter-like conditions. After each acclimation, we measured leukocyte RTL, baseline and stress-induced cortisol (CORT) concentration, and testosterone (T) concentration. Cortisol is the main stress glucocorticoid in Siberian hamsters [52]. We used different animals for RTL and CORT/T measurements. We are aware of the limitations of our experimental setup, namely that CORT and RTL assays were performed in different animals. The first reason for this approach was the need to measure CORT levels during the peak of the winter response, i.e. after 16 weeks of acclimation [53], while RTL was measured after the entire torpor season. The second reason was an ethical issue. Only a limited amount of blood could be taken because it was collected from the retroorbital sinus. We also could not exclude the possibility that any additional bleeding could affect RTL.

Because low *T*_a_ can be perceived as stress ([39]; reviewed in [40]), we used baseline CORT in winter-acclimated animals as a marker of long-term stress. For short-term stress response, hamsters were restrained for 7 min in a custom-made immobilizer in a forced on-back position, which simulated a subordinate social posture [54]. Each restrainer was individually adjusted to animal size to preclude movement.

### (b) Ethical note

All experiments received ethical approval from the Local Committee for Ethics in Animal Research in Bydgoszcz, Poland (decision no. 56/2022).

### (c) Animals and housing

Siberian hamsters (*Phodopus sungorus*) originated from our outbred colony, which is kept under changing photoperiodic conditions of 16 h of light during summer and 8 he of light during winter. We used 230 adult hamsters of both sexes: 120 (55 males and 65 females) for relative telomere length study (‘RTL group’) and 110 animals (55 males and 55 females) for cortisol and testosterone measurements (‘CORT/T group’).

All hamsters were born under summer-like conditions (long photoperiod, 16L : 8D, 20°C). At the age of 3−4 months, part of them (33 females and 27 males in the ‘RTL group’, and 28 females and 26 males in the CORT/T group) were transferred to stable winter-like conditions (8L : 16D, 7°C), while the other part (32 females and 28 males in the RTL group, and 27 females and 29 males in the CORT/T group) were moved to unstable winter-like conditions (8L : 16D, *T*_a_ varying between 7 and 20°C in an unpredictable cycle; electronic supplementary material, figure S1). Thus, the mean temperatures during the entire winter acclimation were 7.9°C under stable conditions and 11.3°C under unstable conditions.

Under both summer and winter acclimation regimes, hamsters were housed in standard laboratory cages (33 × 20 × 18 cm high) with wood shavings, paper tubes and paper towels for bedding and nesting material; food (standard rodent diet; Labofeed B, Morawski, Kcynia, Poland) and water were available ad libitum. Before acclimation to winter, all animals were implanted subcutaneously with thermosensitive PIT-Tags (BioTherm 13, Biomark, Boise, ID, USA) to show their ID and monitor body temperature (*T*_b_). During acclimation to a short photoperiod, *m*_b_ and fur colour were measured every two weeks. A hamster was classified as a responder if its fur changed to white (at least stage 2 or 3 of winter fur according to the Figala scale [55]) and if it used daily torpor (at least one episode). Torpor occurrence was assessed using *T*_b_ measurements (*T*_b_ <30°C). Body temperature was measured once daily, at different times on different days, with a remote reader (HPR plus, Biomark, Boise, ID, USA). This allowed us to gather information about the torpor frequency but not the length or depth of torpor episodes.

### (d) Blood collection

Blood (100 μl) was collected with heparinized haematocrit capillaries from the retroorbital sinus under isoflurane anaesthesia. In the CORT/T experiment, blood was centrifuged for 10 min at 12000*g* and 4°C, and plasma was frozen at −20°C until analysis. For telomere analysis, whole blood was frozen. After blood collection, a drop of analgesic, 0.5% Alcaine solution (proxymetacaine hydrochloride, Alcon, Belgium), was applied to the eye. Blood was drawn twice: after summer-like acclimation in all animals (3–4 months old), and then after 16 weeks of winter-like acclimation for cortisol and testosterone assays, or at the end of winter-like acclimation (22 weeks) for telomere length assays. During the last bleeding, a small drop of blood was also taken with non-heparinized capillaries for blood smears (to analyse the leukocyte profile). To avoid stress response during measurements of baseline cortisol concentration, the time between picking up the hamster and blood collection was less than 1 min.

### (e) Cortisol and testosterone concentrations

Cortisol and testosterone concentrations were measured using liquid chromatography/tandem mass spectrometry (LC-MS-MS) with a Shimadzu UHPLC Nexera XR system equipped with a binary pump coupled with a triple-quadrupole mass spectrometer (LCMS-8045, Shimadzu Corporation, Kyoto, Japan). We used cortisol-D4 as an internal standard, and for testosterone measurements, we did a calibration curve within a range from 0.5 to 8 ng ml^−1^. Data collection, peak integration and calculations were performed using the LabSolutions LCMS software (Shimadzu Corporation, Kyoto, Japan). Details of the procedure are available in the electronic supplementary material.

### (f) Leukocyte profile analysis

To control for the potential effect of age-related changes in leukocyte profiles or any inflammation process on RTL, we did blood smears to count the number of different leukocyte cells. Blood smears were stained using a Leica ST5020 Multistainer (Leica Biosystems, USA). All leukocytes were counted by examining blood smears under 1000× magnification using an oil immersion objective (Olympus UIS2, PlanC N, 100×/1.25 Oil FN22) with an Olympus CX21 microscope (Tokyo, Japan). The proportion of different leukocyte types was obtained by examining 100 leukocytes on each slide and classifying them based on morphology as lymphocytes, neutrophils, eosinophils, basophils or monocytes ([56,57]; reviewed in [58]). Neutrophil to leukocyte (N : L) ratio was also used as a marker of stress. Detailed procedure and the proportion of white blood cells are available in electronic supplementary material, table S2.

### (g) Telomere length analysis

Genomic DNA was extracted from heparinized whole blood using the Extractme Genomic DNA Kit (CytoGen), according to the manufacturer’s protocol. RTL was measured by quantitative polymerase chain reaction (qPCR) [59]. The principle underlying this method is that the amount of telomere signal detected per genome through qPCR correlates with the average telomere length within a given DNA sample. RTL was calculated from non-baseline corrected raw data by means of the LinRegPCR software, using a formula described previously in Ruijter *et al*. [60]. The mean qPCR efficiency was determined using the amplification plot method [61]. See the electronic supplementary material for a detailed description of this procedure.

### (h) Statistical analysis

All statistical procedures were done in R v. 4.2.3 [62]. Seasonal changes in body mass, cortisol level, telomere length, lymphocyte proportion and testosterone level were analysed with linear mixed-effects modelling (LME; package lme4 [63]). We also tested the effect of winter conditions and phenotype on Δtelomeres and Δlymphocytes (calculated as the difference between winter and summer acclimation) with general linear models (GLMs; package stats [62]). The model for Δtelomere also included telomere length in summer. A statistical artefact may occur when regressing a difference between two measurements, here RTL1 (RTL after summer acclimation) and RTL2 (RTL after winter acclimation), against either variable RTL1 or RTL2, known as regression to the mean (RTM) [64]. Since this artefact was also observed in telomere length dynamics studies [65], we reanalysed data using a correction to avoid it (equations are available in the electronic supplementary material). A GLM was also used to test the effect of torpor frequency on Δtelomeres in responding individuals.

Initial models included season (winter, summer), winter conditions (stable or unstable), phenotype (responder, non-responder) and sex as independent factors, body mass as a covariate, and animal ID as a random factor in mixed models. Initial models were full models; hence they included all fixed factors and all possible interaction terms. Insignificant terms were removed from the model using stepwise backward elimination (when *p* > 0.05). We obtained a series of models that were compared, and the final models were chosen using the Akaike information criterion (AIC; package MuMIn [66]), to see if removing any insignificant term did not reduce the fitting of the model. We chose models with the lowest AIC, which were also the simplest ones. All differences were considered significant at *p* ≤ 0.05. The type III variance analysis results are provided in electronic supplementary material, tables S3 and S4. All results are presented as estimated marginal means ± s.e. (package emmeans [67]).

#### (i) Final models for cortisol/testosterone assays

The model for seasonal changes in body mass included all fixed factors (four) and all second-order interactions except phenotype × winter conditions (five), plus third-order interaction of winter conditions × sex × season (electronic supplementary material S1, table S3). The model for seasonal changes in basal cortisol level included all fixed factors (four) and body mass as a covariate. It also included three interactions: season × body mass, season × sex and winter conditions × season. The model for seasonal changes in cortisol scope included the same fixed factors but only two interactions: phenotype × season and winter conditions × phenotype (electronic supplementary material, table S3). Cortisol scope was calculated by dividing short-term stress-induced cortisol concentration by baseline cortisol level. Since seasonal changes in testosterone concentration were analysed only in male hamsters, the analysis included only three fixed factors and body mass. The final model did not include any interaction. To examine the relationship between cortisol concentration and testosterone level, we added testosterone level to the baseline cortisol model as a covariate. The final model included only the main factors (electronic supplementary material, table S3).

#### (ii) Final models for telomere assay

The model for seasonal changes in body mass included all fixed factors (four) and the interaction of phenotype and season. The model for seasonal changes in RTL included all fixed factors (four), body mass as a covariate and interaction of winter conditions and season (electronic supplementary material S1, table S3). The model for Δtelomeres included the effect of phenotype, winter conditions, sex, Δbody mass and telomere length in summer and the interaction of phenotype and telomere length in summer. We tested the effect of torpor use on Δtelomeres only in responders, so the final model included only sex and winter conditions as fixed factors, and also Δbody mass, telomere length in summer and torpor occurrence. The model for seasonal changes in lymphocyte proportion included all fixed factors (four) and body mass and the interaction of sex and body mass, while the model for Δlymphocytes included only the effect of phenotype, winter conditions and sex (electronic supplementary material S1, table S4). The final model for the N : L ratio included only fixed factors and a covariate (electronic supplementary material S1, table S4). The number of lymphocytes was taken to analyse the potential effect of inflammation on RTL changes. To examine the relationship between RTL and lymphocyte proportion, we used LME, and to test the covariation between Δtelomeres and Δlymphocytes, we used GLM. We used telomere as the dependent variable and lymphocyte proportion as a covariate. To avoid autocorrelation, the models did not include body mass, which correlated with the lymphocyte proportion. The model for the relationship between RTL and lymphocyte proportion included all four fixed factors, lymphocyte proportion as a covariate, and interaction: winter conditions × season. The model for the relationship between Δtelomeres and Δlymphocytes included three fixed factors (winter conditions, phenotype, sex) and Δlymphocytes.

## 3. Results

### (a) Seasonal changes in cortisol and testosterone level

**Figure 1.**
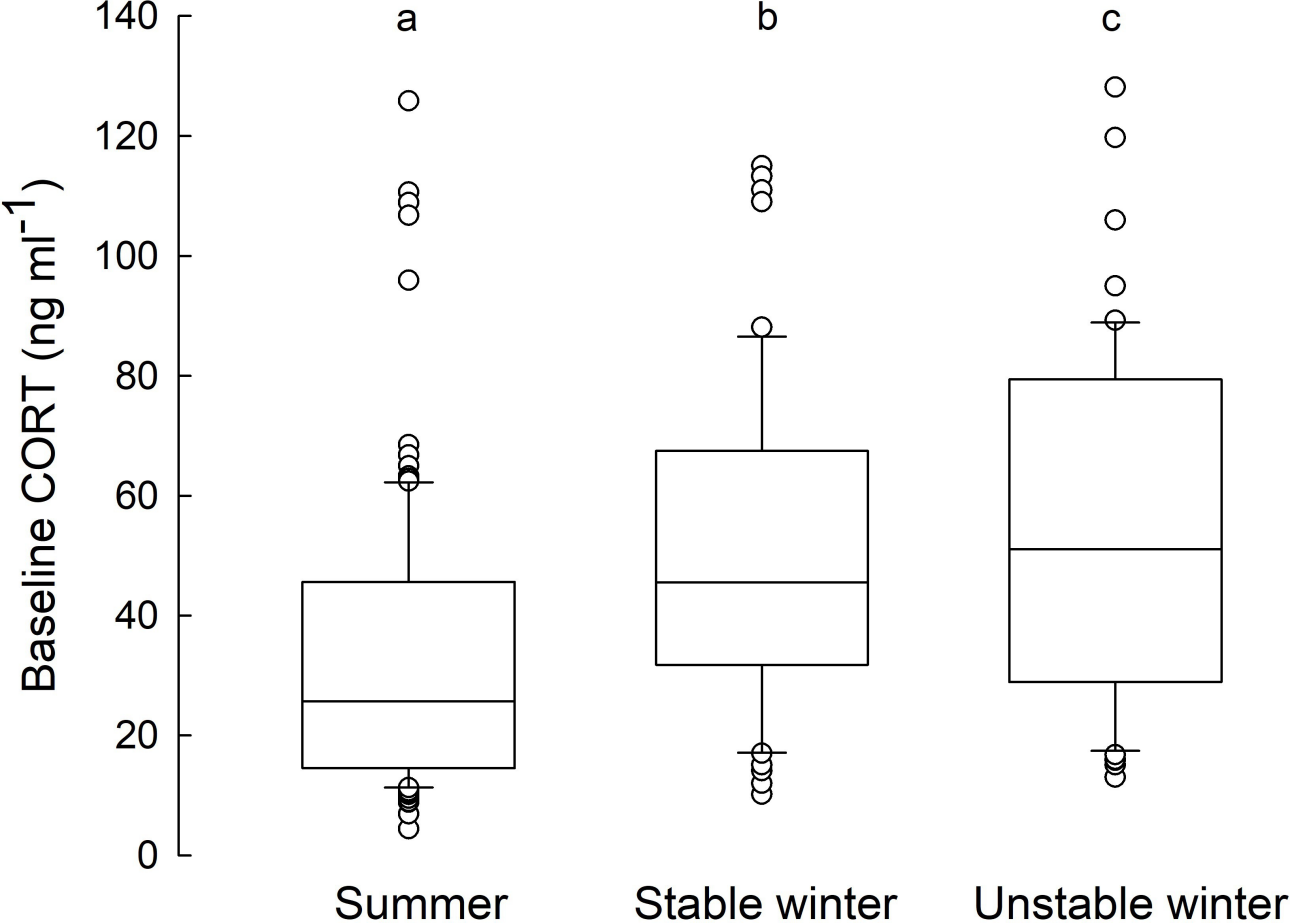
Baseline cortisol (CORT) concentration (ng ml^−1^) in the Siberian hamsters acclimated to summer (*n* = 110), stable winter (*n* = 54) or unstable winter conditions (*n* = 56). Data obtained from responders and non-responders were pooled together because phenotypes did not differ. Horizontal lines within boxes indicate medians, boxes cover the 25th to 75th percentiles, whiskers indicate the 10th and 90th percentiles, and dots indicate outliers. Statistically significant differences are indicated with lowercase letters: a–b, a–c: *p* < 0.001; b–c: *p* = 0.031).

In winter, hamsters kept under unstable conditions had higher baseline CORT than hamsters kept under stable conditions (53.8 ± 3 vs 44.5 ± 3.2 ng ml^−1^), while in summer CORT levels were similar (33.2 ± 3.1 vs 35.1 ± 3 ng ml^−1^, figure 1; season × winter conditions: *F*_1,106_ = 4.8, *p* = 0.031). Baseline CORT was related to sex, season and body mass but not to winter phenotype. It was higher in winter than in summer (49.56 ± 2.19 vs 34.13 ± 2.19 ng ml^−1^; *F*_1,123_ = 13.9, *p* < 0.001) and in females than in males (47.4 ± 2.6 vs 35.9 ± 2.6 ng ml^−1^; *F*_1,125_ = 9.3, *p* = 0.003). However, sex differences were recorded only in summer (season × sex: *F*_1,110_ = 4.8, *p* = 0.030), when the CORT level in males was markedly lower (25.1 ± 3.4 ng ml^−1^). Finally, the smaller the hamster, the higher its baseline CORT concentration, and this relationship was stronger in winter than in summer (season × body mass: *F*_1,123_ = 8.6, *p* = 0.004). Detailed data on seasonal body mass changes are available in electronic supplementary material S1, results and table S1).

**Figure 2.**
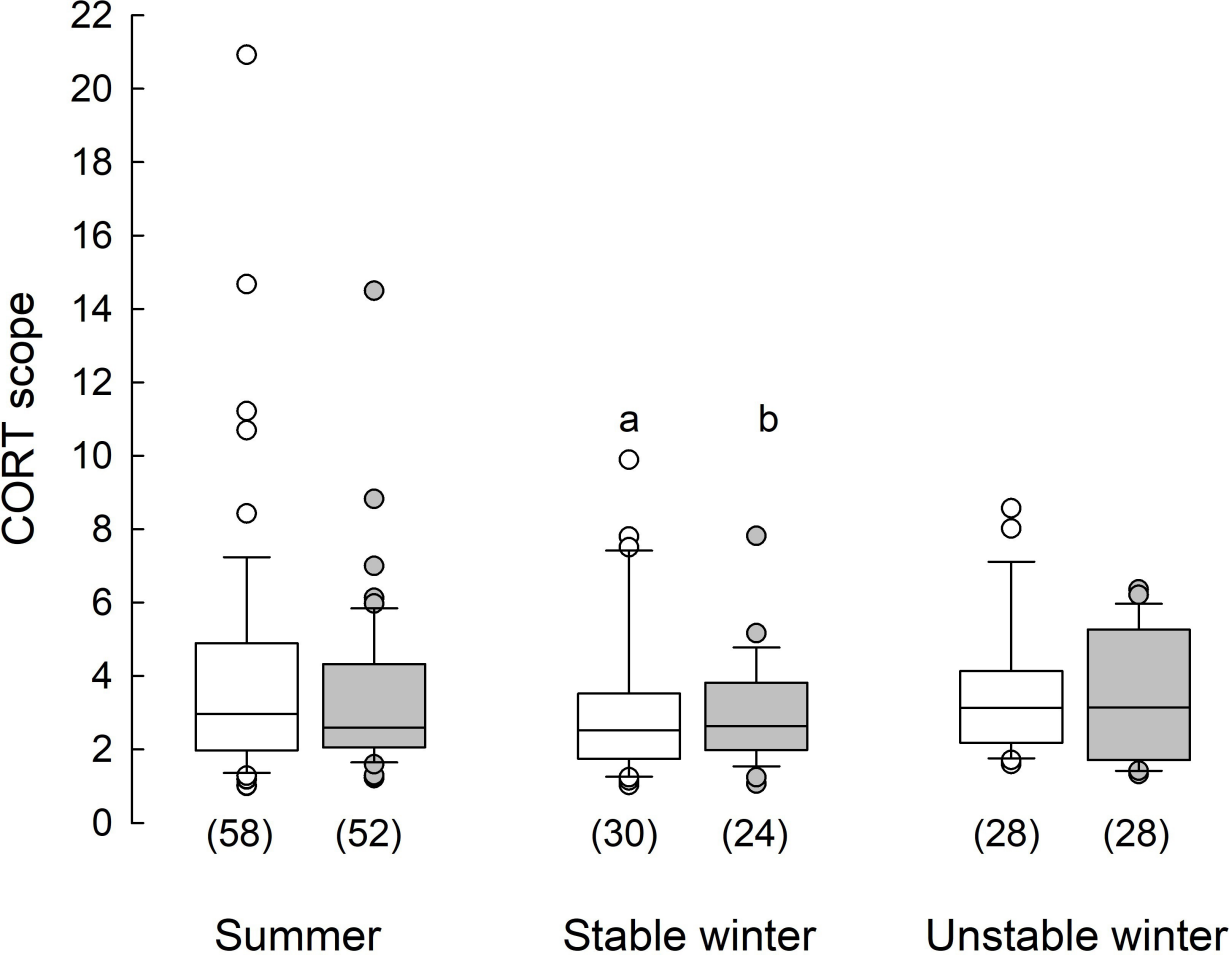
Cortisol scope in responders (white boxes) and non-responders (grey boxes) acclimated to summer and stable or unstable winter conditions. Numbers in parentheses stand for each group size. Horizontal lines within boxes indicate medians, boxes cover the 25th to 75th percentiles, whiskers indicate the 10th and 90th percentiles, and dots indicate outliers. The statistically significant difference between phenotypes is indicated with lowercase letters: a–b: *p* = 0.036).

Non-responders had higher CORT scope than responders only in winter (4.0 ± 0.3 and 2.7 ± 0.4, phenotype × season: *F*_1,157_ = 9.6, *p* = 0.002), and only under stable winter conditions (4.1 ± 0.4 and 3.1 ± 0.3, respectively, winter conditions × phenotype: *F*_1,104_ = 4.5, *p* = 0.036; figure 2). Under unstable conditions CORT scope was similar (non-responders 3.31 ± 0.34 and responders 3.77 ± 0.33). Cortisol scope was not related to body mass (*F*_1,146_ = 0.6, *p* = 0.454), but it was always higher in males than females (4.2 ± 0.3 vs 2.9 ± 0.3; *F*_1,114_ = 10.9, *p* = 0.001).

**Figure 3.**
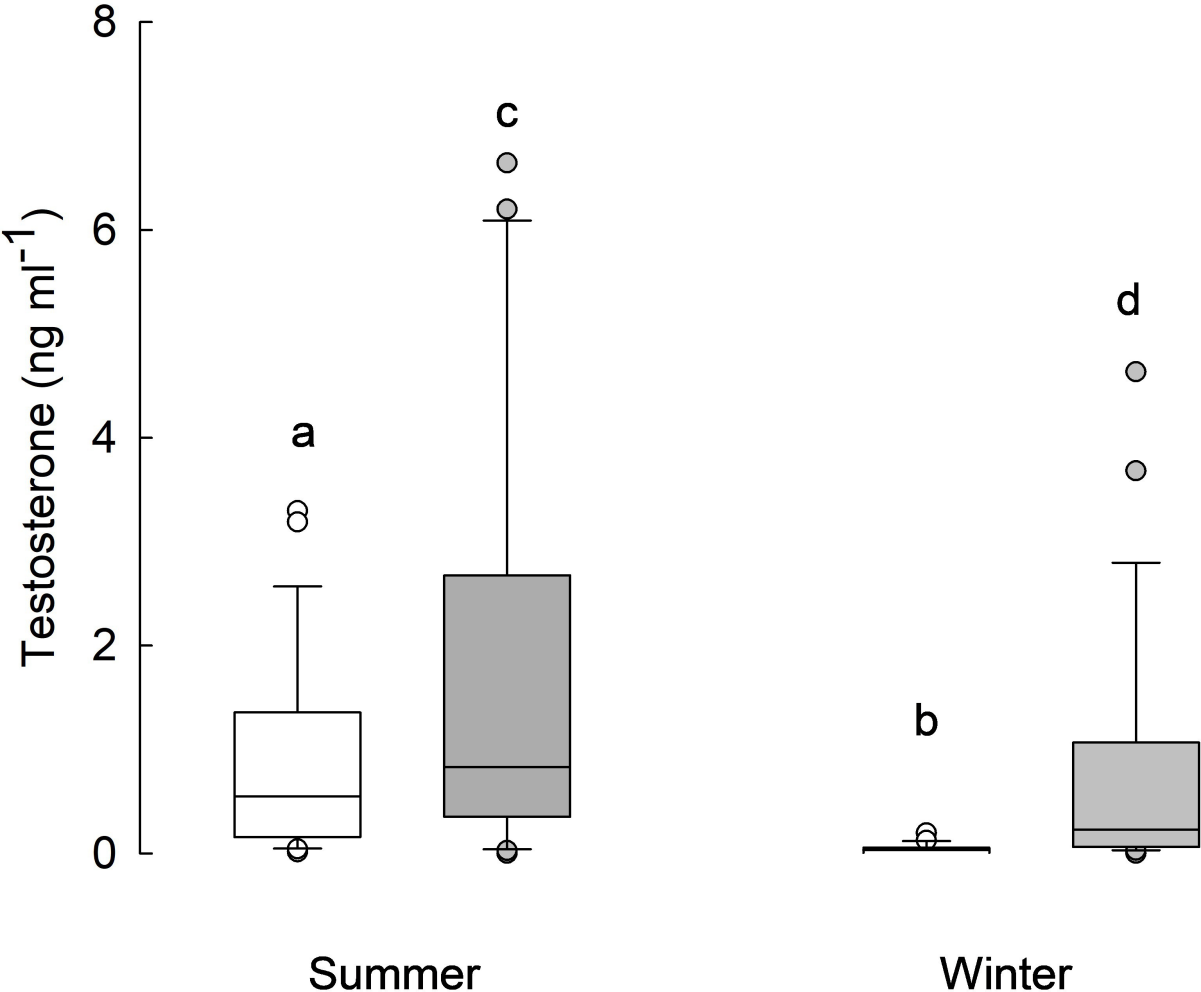
Testosterone concentration (ng ml^−1^) in male responders (white boxes, *n* = 29) and non-responders (grey boxes, *n* = 26) in summer and winter. Horizontal lines within boxes indicate medians, boxes cover the 25th to 75th percentiles, whiskers indicate the 10th and 90th percentiles, and dots indicate outliers. Statistically significant differences are indicated with lowercase letters: a–b, c–d: *p* = 0.007; a–c, b–d: *p* = 0.02).

Testosterone concentration in males was related to season (*F*_1, 58_ = 7.7, *p* = 0.007) and phenotype (*F*_1, 60_ = 5.7, *p* = 0.020; figure 3), but not to winter conditions (*F*_1, 51_ = 2.5, *p* = 0.116), body mass (*F*_1, 99_ = 0.03, *p* = 0.854), and cortisol concentration (*F*_1, 104_ = 0.01, *p* = 0.911). Testosterone concentration was lower in winter (0.5 ± 0.2 ng ml^−1^) than in summer (1.3 ± 0.2 ng ml^−1^), and it was always higher in non-responders (1.3 ± 0.2 ng ml^−1^) than in responders (0.5 ± 0.2 ng ml^−1^).

### (b) Seasonal changes in relative telomere length (RTL) and lymphocyte proportion

**Figure 4.**
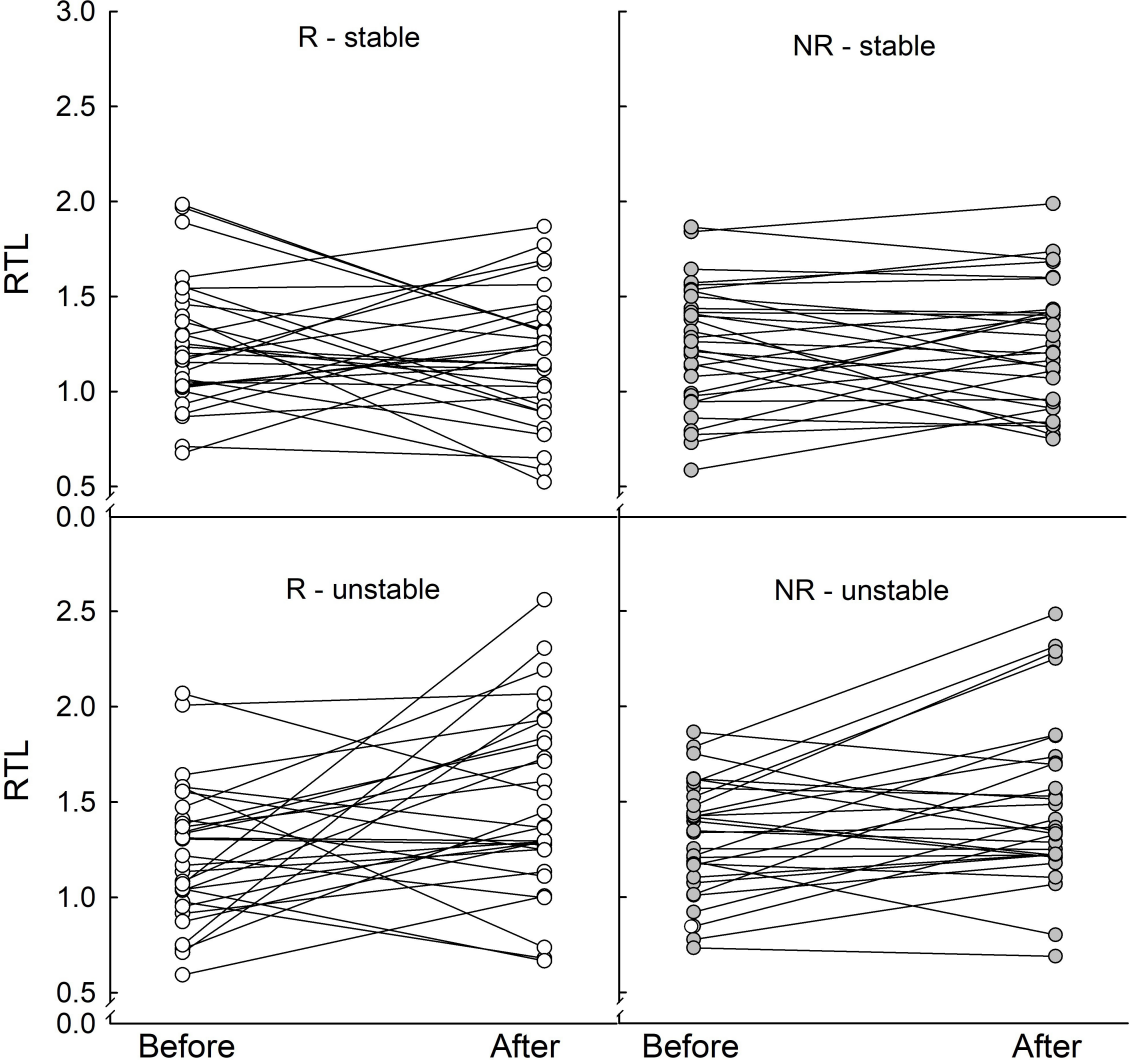
Individual changes in the relative telomere length (RTL) in responders (R, open symbols) and non-responders (NR, grey-filled symbols) before and after acclimation to stable or unstable winter conditions. Data points represent 30 individuals in each group.

RTL depended on season and winter conditions (season × winter condition: *F*_1, 117_ = 10.9, *p* = 0.001; figure 4). Under unstable winter conditions, telomeres were longer after winter than before it (1.49 ± 0.05 and 1.26 ± 0.05, respectively), while under stable winter conditions, RTL did not change significantly (1.23 ± 0.05 before and 1.21 ± 0.05 after winter). As a result, change in RTL (Δtelomeres) depended on winter conditions, being larger under unstable than under stable conditions (*F*_1, 113_ = 13.9, *p* < 0.001).

**Figure 5.**
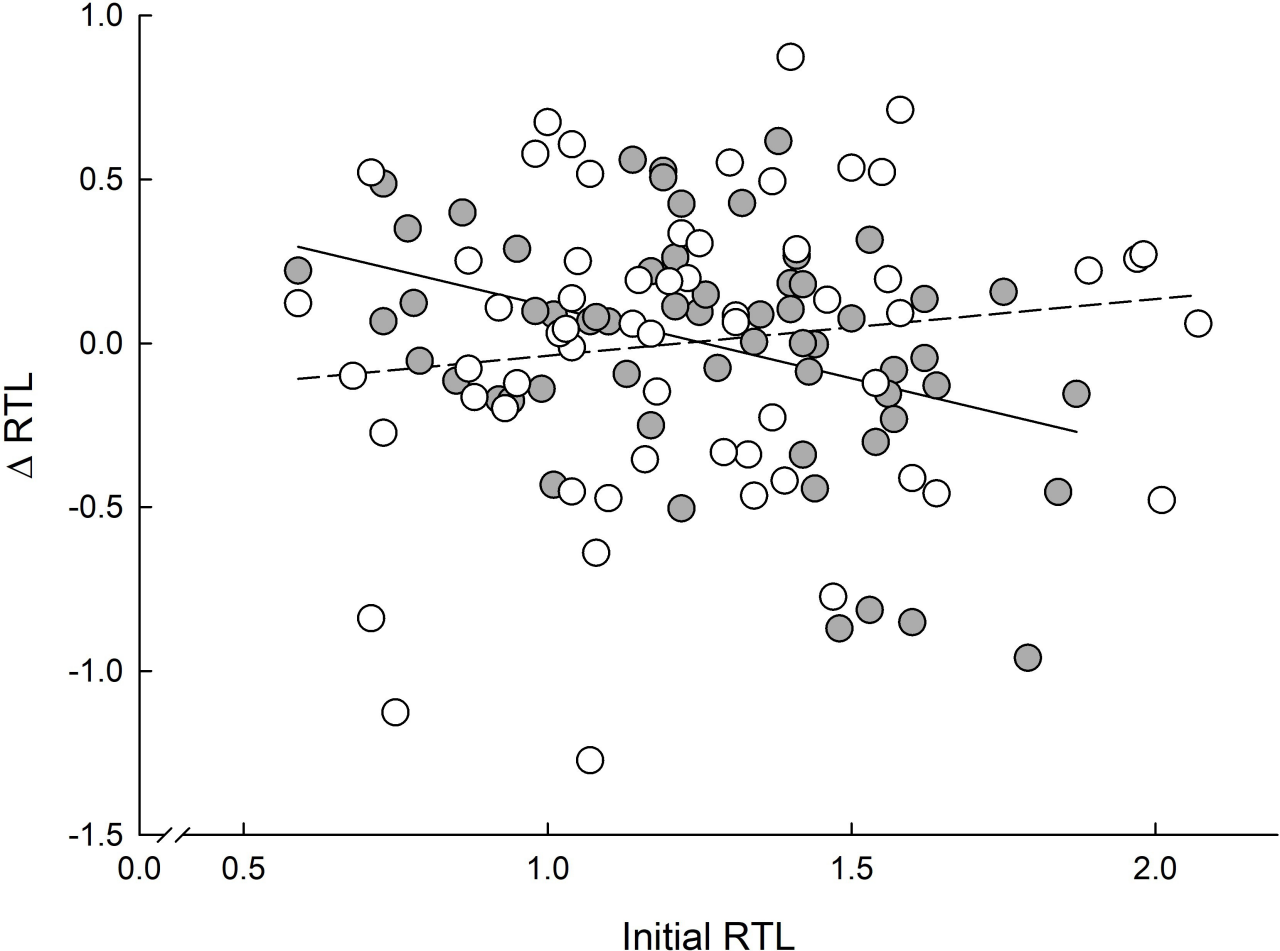
The relation between initial (summer) RTL and seasonal change in RTL (ΔRTL adjusted for the regression to the mean) in Siberian hamsters responding (open symbols, dashed line; *y* = 0.17 (0.17)*x* − 0.21 (0.21)) and non-responding (grey-filled symbols, solid line; *y* = −0.44 (0.14)*x* + 0.55 (0.18)) to short photoperiod. Data obtained from animals housed under different winter conditions (stable and unstable) were pooled together because there was no difference between the groups.

Change in telomere length (Δtelomeres) was related to phenotype (0.12 ± 0.05 in non-responders and 0.06 ± 0.05 in responders) and to initial (summer) telomere length but only in non-responders (summer telomere length × phenotype: *F*_1, 113_ = 6.4, *p* = 0.012; figure 5). The relationship between torpor frequency and seasonal changes in RTL was strong and positive (*F*_1, 54_ = 4.8, *p* = 0.033), with a higher incidence of torpor bouts resulting in a larger increase in RTL (*y* = 0.017 (0.001)*x* − 0.42 (0.16)).

Lymphocyte proportion did not depend on the season (*F*_1, 209_ = 2.8, *p* = 0.091), phenotype (*F*_1, 115_ = 1.2, *p* = 0.276) or winter conditions (*F*_1, 115_ = 0.9, *p* = 0.354; electronic supplementary material, table S4), but it was related to body mass and sex (sex × *m*_b_: *F*_1, 215_ = 3.9, *p* = 0.048). Lymphocyte proportion decreased with increased body mass, but only in males. At the same time, seasonal changes in the proportion of lymphocytes (Δlymphocytes) depended on winter phenotype (*F*_1, 116_ = 8.5, *p* = 0.004). It decreased by approximately 3% in non-responders and increased by approximately 1% in responders (electronic supplementary material, table S4). We did not find a relationship between lymphocyte proportion and telomere length (*F*_1, 224_ = 0.1, *p* = 0.811) or Δtelomeres and Δlymphocytes (*F*_1, 115_ = 0.01, *p* = 0. 115). Other types of leukocytes also did not affect RTL changes (electronic supplementary material, table S5).

The neutrophils to lymphocytes (N : L) ratio did not depend on season (*F*_1, 124_ = 1.20, *p* = 0.275), phenotype (*F*_1, 120_ = 1.35, *p* = 0.247), or winter conditions (*F*_1, 115_ = 1.44, *p* = 0.232).

## 4. Discussion

Low ambient temperature and reduced food availability in winter evoke energetic stress responses manifested by increased glucocorticoid concentration ([39]; reviewed in [40]). Our results indicate that low ambient temperature in winter increased baseline CORT concentration independent of phenotype and that warm spells augmented stress response (figure 1). However, hamsters exposed to warm spells had longer telomeres after winter acclimation than individuals maintained in constant cold. Among responders, more frequent torpor resulted in a larger increase in RTL.

Seasonal differences in baseline CORT can be explained by the ‘energy mobilization’ hypothesis, which posits that GC levels are high at the time of high energy demands [2]. Low ambient temperature poses an energetical challenge that requires a higher metabolic rate to maintain a constant body temperature. The meta-analysis of data on GC and metabolic rate also showed that the variations in metabolic rate are a key factor driving variations in GC levels [3]. In the context of reproduction, the higher CORT concentration in winter would support the 'inhibitory hypothesis’, suggesting that high GCs suppress breeding [68]. It was also proposed that seasonal breeders should be more resistant to social and environmental stress (which inhibit reproduction) because of their shorter breeding seasons [69]. Our results do not support these hypotheses. We observed increased baseline CORT in winter in both phenotypes, while only non-responding individuals do not reduce gonads [27] and thus can breed in winter or commence breeding season earlier than responders [30]. Independent of the season, the smaller the hamster, the higher the baseline CORT that was recorded. This agrees with the hypothesis that baseline cortisol concentration is linearly related to mass-specific metabolic rate [70]. Body mass can also explain higher baseline CORT in females than males. Smaller Siberian hamsters are also bolder and more prone to take risks than bigger ones because of the higher resource acquisition costs [71]. Increased baseline CORT in winter may also be related to higher aggressiveness [72–75], which results from harsh environmental conditions and limited resources [72], not from a higher concentration of gonadal steroids [74,76–78]. Our data show that CORT level was not related to testosterone concentration. Both phenotypes had significantly lower testosterone concentrations in winter than in summer (figure 3). Interestingly, the winter testosterone level in non-responders was as high as the summer level in responders. This may suggest that very high testosterone concentration in summer determines phenotype development towards the non-responding one. This assumption is based on the fact that testosterone inhibits daily torpor, the main trait of winter phenotype in responding individuals [79–81].

Like baseline GC, stress-induced GC concentration may also show annual rhythm [2,41]. Under stable winter conditions, non-responders had a larger CORT scope than responders (figure 2). Many studies showed that the response to short-term stress is higher in short-day animals than in long-day individuals [82–84], but there is also a study showing a similar CORT response under both photoperiod regimes [85]. Unfortunately, data are available only for responders. We suggest that the difference in CORT scope between phenotypes under stable winter conditions implies higher environmental pressure on non-responders, which would be favoured in an unpredictable environment. A winter decrease in the proportion of lymphocytes in non-responders also indicates that a cold winter is more energetically demanding for them than for responders. Higher testosterone concentration can also suppress the immune system ([86,87]; but see [88]). Moreover, the winter differences between phenotypes in CORT scope may also result from differences in the basal metabolic rate (BMR). Contrary to responders, non-responders increase BMR from summer to winter [43].

We predicted that increased GC levels may lead to a faster telomere attrition rate ([4,89]; reviewed in [5]). Because telomeres shorten faster in more demanding conditions ([9]; reviewed in [5]), we predicted that an unpredictable, stressful environment, i.e. warm spells in winter, results in shorter telomeres. However, the opposite was true. We found that RTL increased under unstable winter conditions, independent of phenotype, but did not change in stable cold (figure 4). This may indicate the positive effect of a mean warmer temperature (which requires lower energy expenditure) on RTL in animals exposed to warm spells in winter. Telomere lengthening may indicate higher telomerase activity in warmer ambient temperatures, suppressing the negative effect of CORT ([90]; reviewed in [5]). Among white blood cells, telomerase can be found only in lymphocytes [91]. In T-cells and B-cells telomerase activity is highly expressed during development, markedly reduced in the resting lymphocytes, and then rapidly up-regulated upon activation, e.g*.* during stress [92]. In our study, ΔRTL did not depend on seasonal changes in the proportion of lymphocytes (Δlymphocytes). However, Δlymphocytes depended on winter phenotype, decreasing in non-responders by approximately 3% and increasing in responders by approximately 1%. The magnitude of these changes may seem small, but the calculated effect size (Cohen’s *d *= 0.39) indicates the significant importance of this difference.

Because RTL over the winter did not change or even increase, we propose that food availability and the mean warmer temperature might allow better performance in the laboratory than in the wild. Under natural conditions, when energy resources are limited, and animals have to cope with other stressors, telomere elongation may not be possible. A similar conclusion was drawn earlier, in studies on hibernating animals ([45,46]; reviewed in [47]). Small mammals can restore telomere length during the active season [45] because telomerase is active not only in their germline but also in somatic tissues [50,93,94]. The rate of telomere shortening was related to the periodic arousals from hibernation, the rate of rewarming and the time spent euthermic [45,50,95,96]. While in hibernating bats, telomerase activity may be higher than during normothermia ([97]; reviewed in [98]), in the hibernating garden dormouse (*Eliomys quercinus*), telomerase is present during torpor but its activity is markedly reduced [99]. There is much evidence that oxidative stress during arousal accelerates telomere shortening [89,94,100]. The higher the difference between the body and ambient temperatures, the higher the energy cost of arousal and oxidative stress. Thus, animals hibernating at higher ambient temperatures can maintain longer telomeres than individuals hibernating at lower *T*_a_ [46]. After hibernation or during interbout euthermia, telomere length can be restored if food is abundant [45,46,101,102]. The positive effect of food availability on RTL gave rise to the ‘metabolic telomere attrition’ hypothesis, which posits that the RTL dynamics are linked to an energetic trade-off, with telomeres shortening during energy shortages [13]. The energetic costs of arousal could be a key driver of the RTL dynamics (reviewed in [47]). We predicted that non-responders would have shorter telomeres than responders because they do not use daily torpor, which retards telomere shortening [44]. However, the opposite was true. RTL increased by 0.12 ± 0.05 in non-responders and only by 0.06 ± 0.05 in responders. This does not indicate that daily torpor shortens RTL, because ΔRTL was positively related to torpor frequency among responders. In a previous experiment on Siberian hamsters, changes in RTL were best explained by the positive effect of daily torpor frequency, changes in body mass and initial RTL [44]. In our study, initial RTL (approx. 1.2) was the same as reported by Turbill and co-workers [44], but the correlation between initial RTL and ΔRTL was recorded only among non-responders (figure 5). The difference in ΔRTL between phenotypes cannot be explained by the effect of oxidative stress, which is similar in individuals responding and non-responding to short photoperiod [31]. This may be related to the fact that returning to normothermia from daily torpor does not induce significant oxidative stress, as it is far less energetically demanding than returning to normothermia from hibernation. The results of studies on animals hibernating at higher temperatures support this idea. In a tropical primate, the fat-tailed dwarf lemur (*Cheirogaleus mediu*s), hibernating at 11–15°C, telomeres lengthened during hibernation season. In black bears (*Ursus americanus*), which hibernate at much higher body temperature than small animals, RTL increases with the duration of the hibernation period [103]. Also, in young European badgers (*Meles meles*), animals experiencing lower temperatures during the first spring had shorter telomeres than cubs at warmer temperatures [104]. All the above examples strengthen the ‘metabolic telomere attrition’ hypothesis.

## 5. Concluding remarks

Appropriate adjustments to the environment increase animal fitness. Central climate change theory predictions emphasize an increase in mean ambient temperature and an increased probability of extreme hot events [105]. Increased variations in *T*_a_, rather than increased mean *T*_a_, shape population responses to global climate change [106]. On the one hand, higher temperatures in winter reduce energy expenditure and allow telomere lengthening. On the other hand, daily torpor retards telomere shortening. Warm spells in winter may induce more frequent arousals, which can be detrimental unless animals can gain sufficient resources. Both phenotypes, responding and non-responding to short photoperiod, increase baseline cortisol levels in winter, which (if it remains within the homeostatic range) allows them to function better under stress and prepare for subsequent stress responses (reviewed in [107]). However, in non-responders, cortisol scope was higher under stable than unstable winter conditions, indicating that this phenotype is more vulnerable to stress under constant cold. The decreased proportion of lymphocytes in non-responders during winter supports this suggestion. Responding and non-responding individuals occur in the same population, suggesting that, despite phenotypic differences, they overcome the challenges posed by environment. If the future extinction rate depends on the magnitude of environmental changes, phenotypic flexibility may buffer the population from global climate change.

## Data Availability

Data are publicly available on Dryad [108]. Supplementary material is available online [109].
